# Case report: The first case of concurrent breast myeloid sarcoma and borderline phyllodes tumor with malignant features

**DOI:** 10.3389/fonc.2023.1268617

**Published:** 2024-01-19

**Authors:** Li Chun, Zhen Zeng, Qianyu Guo, Yangjun He

**Affiliations:** ^1^ Department of Integrative Oncology (Rehabilitation Technology), Sichuan Nursing Vocational College, Chengdu, China; ^2^ Department of Thoracic Surgery, West China Hospital, Sichuan University, Chengdu, China; ^3^ Department of Urology, Feinberg School of Medicine, Northwestern University, Chicago, IL, United States; ^4^ Robert H. Lurie Comprehensive Cancer Center, Northwestern University, Chicago, IL, United States; ^5^ Department of Breast Surgery, Chengdu Seventh People’s Hospital, Chengdu, China

**Keywords:** breast cancer, myeloid sarcoma (MS), borderline phyllodes tumor with malignant features, neoadjuvant radiotherapy, neoadjuvant chemotherapy

## Abstract

**Background:**

Myeloid sarcoma (MS) is a rare hematological malignancy characterized by the formation of a solid mass of myeloblasts outside the bone marrow, such as in the lymph nodes, skin, or bone. MS may arise *de novo* or concurrently with acute myeloid leukemia (AML), myeloproliferative neoplasm (MPN), or myelodysplastic syndrome (MDS). MS accounts for less than 1% of extramedullary acute myeloid leukemia cases. Phyllodes tumors (PTs) are a rare fibroepithelial breast tumor that can be benign, malignant, or borderline, and account for less than 1% of all breast cancers.

**Case presentation:**

We present a unique case of a 50-year-old woman with both breast MS and borderline PT with malignant features, which presented a diagnostic challenge. The patient initially presented with a mass in her right breast, and the initial fine-needle biopsy revealed the presence of immature myeloperoxidase (MPO)^+^ myeloid cells consistent with MS. Subsequent pathological analysis of tumor tissues after neoadjuvant radiotherapy and chemotherapy showed a borderline PT with malignant features. Following excision of the tumor, the patient experienced a local recurrence, which was also surgically removed. At 8 months post-surgery, the patient remains free of recurrence under close follow-up.

**Conclusion:**

This case highlights the importance of considering the possibility of concurrent malignancies in the differential diagnosis of complex breast masses and underscores the challenges involved in diagnosing and managing such cases. Additionally, we also emphasize the value of neoadjuvant radiotherapy and chemotherapy in MS.

## 1 Introduction

Myeloid sarcoma (MS) is a rare and distinct form of hematological malignancy that is characterized by the extramedullary accumulation of myeloblasts. Also known as chloroma, granulocytic sarcoma, or extramedullary myeloid tumor, MS can manifest in various organs, including the skin, lymph nodes, soft tissues, liver, spleen, and bones. There are three types of MS based on the type of cell and degree of differentiation involved: granulocytic sarcoma (GS) type, primitive mononuclear cell sarcoma type, and triple hematopoietic cell myeloid sarcoma type. MS may arise *de novo* or concurrently with AML, myeloproliferative neoplasm (MPN), or myelodysplastic syndrome (MDS), and can even be the initial presentation of AML or a manifestation of AML recurrence (1). The diagnosis of MS can be clinically challenging. Breast phyllodes tumors are a rare type of breast tumor, comprising only 0.3-1.0% of all breast tumors (2), and are classified into benign, borderline, and malignant subtypes. In this case report, we present a unique case of a female patient diagnosed with concurrent breast MS and borderline phyllodes tumor (borderline PT) with malignant features, which, to our knowledge, has not been previously reported.

## 2 Case presentation

**Figure 1:**
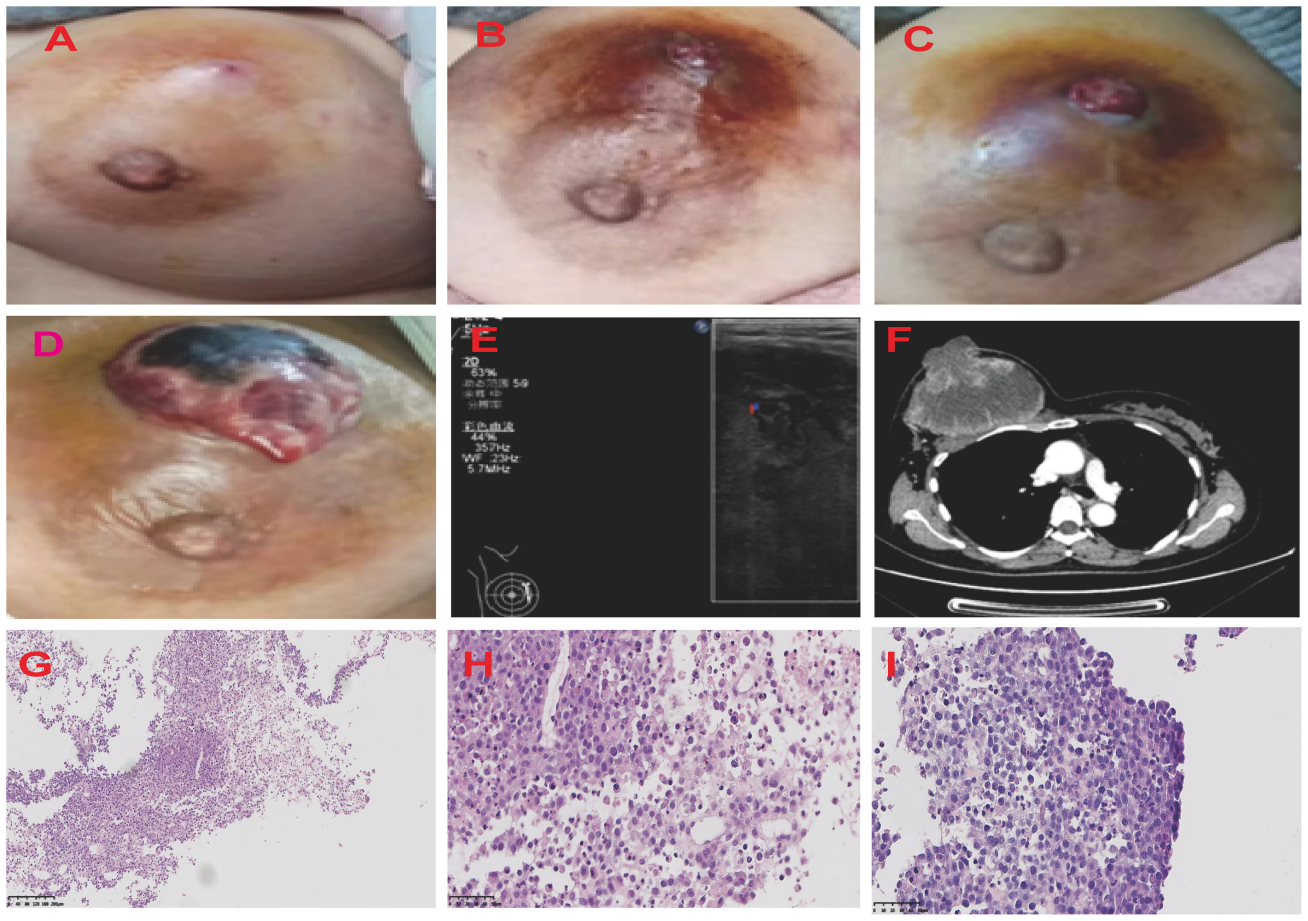
Graphic Representation of Breast Lesion Progression Over Time. The series of four images chronicle the evolution of the right breast lesion from March to April 2021. **(A)** The image of the breast lesion on [5/3/2021]. The patient initially presented with marked breast swelling and tenderness. **(B)** The image of the breast lesion on [5/4/2021]. **(C)** The image of the breast lesion on [23/4/2021]. **(D)** The image of the breast lesion on [25/4/2021]. The breast lesion has evolved into a mass with profound erythema and sinus tract. The ultrasound and CT images of the patient’s right breast **(E, F)**. The H&E staining of MS. **(G)** The description of myeloblasts and lymphoid H&E×100. **(H)** The description of myeloblasts and lymphoid H&E×400. **(I)** The description of tumor cells H&E×400. H&E, hematoxylin and eosin.

A 50-year-old woman presented to the 7th People’s Hospital of Chengdu with an erythematous breast mass that was painful and had central necrosis in the upper outer quadrant of her right breast (**Figures 1A–D**). Physical examination revealed an immobile mass in the upper outer quadrant of her right breast, with an orange-peel appearance, but no signs of nipple retraction were noted. The patient had a medical history of well-controlled hypertension, which was being treated with felodipine and enalapril. She denied any history of smoking, alcohol or substance abuse, and had no family history of cancer. Her physical examination, aside from the breast mass, was unremarkable.

**Figure 2:**
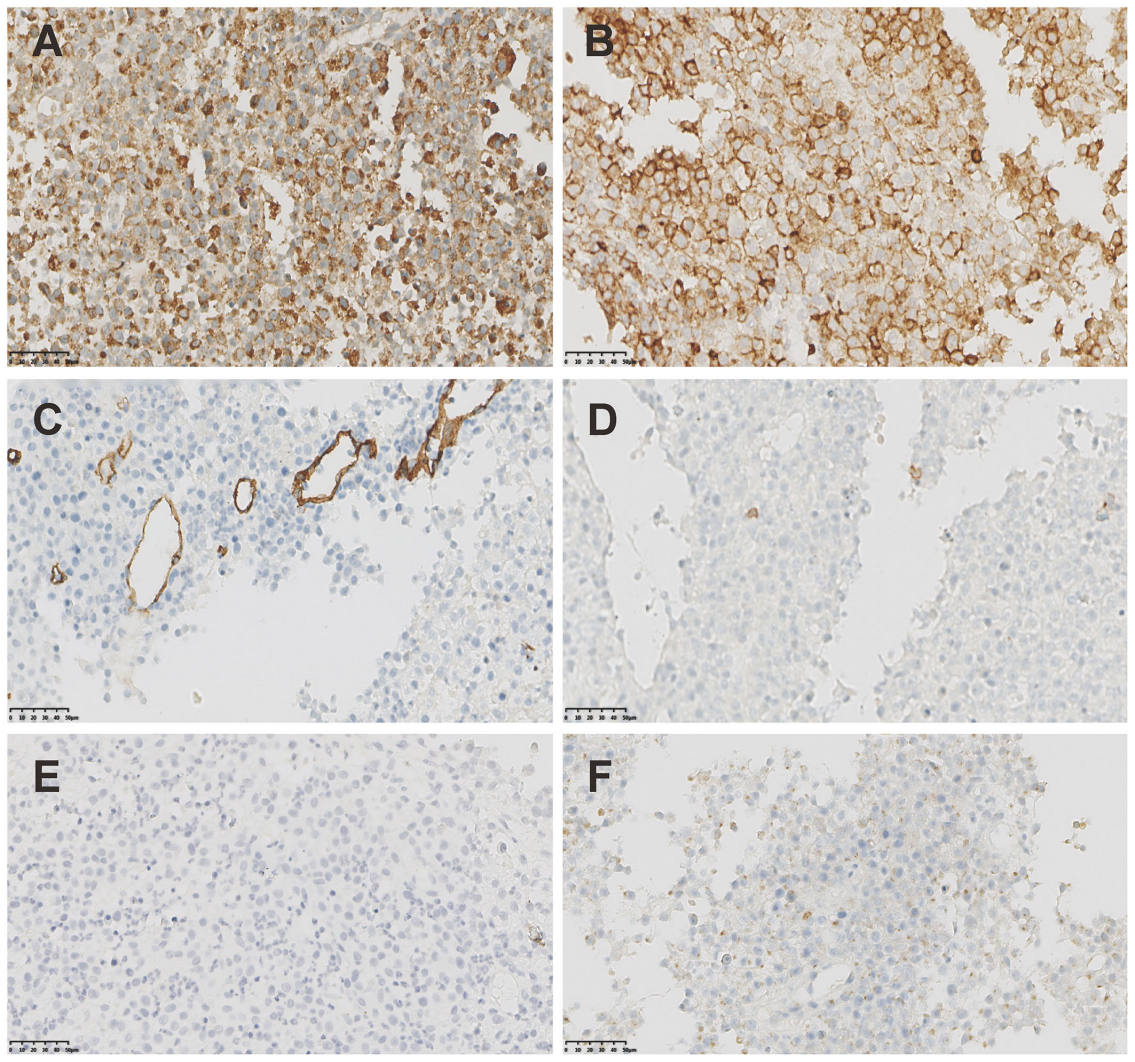
The IHC staining of MS. **(A)** Strong positivity of CD68KP1 (×200). **(B)** Strong positivity of CD45(×200). **(C)** Negative staining of CD34(×200). **(D)** Negative staining of CD3(×200). **(E)** Negative staining of CD20(×200). **(F)** Negative staining of CD79a(×200).

Breast ultrasound revealed an 8.8cm mass with mixed solid echogenic and cystic anechoic components. (**Figure 1E**). The contrast-enhanced CT scan showed an 11.7×8.9cm mass occupying a substantial portion of the right breast, accompanied by right axillary lymphadenopathy (**Figure 1F**). An ultrasound-guided fine needle biopsy revealed the presence of immature myeloid cells (**Figures 1G–I**). Immunohistochemistry (IHC) analysis demonstrated strong expression of CD68KP1 (**Figure 2A**), CD45 (**Figure 2B**), CD33, Cyclin D1 and CD4, with weak positivity for myeloperoxidase (MPO), and a high level of Ki67 expression (80%). The cells were negative for CD34 (**Figure 2C**), CD3 (**Figure 2D**), CD20 (**Figure 2E**), CD79a (**Figure 2F**), CD14, CD117, and terminal deoxynucleotidyl transferase (TdT). A bone marrow biopsy was performed, and there was no evidence of systemic hematological malignancies such as AML, MPD, or MDS. Therefore, the patient was diagnosed with isolated myeloid sarcoma (MS).

**Figure 3:**
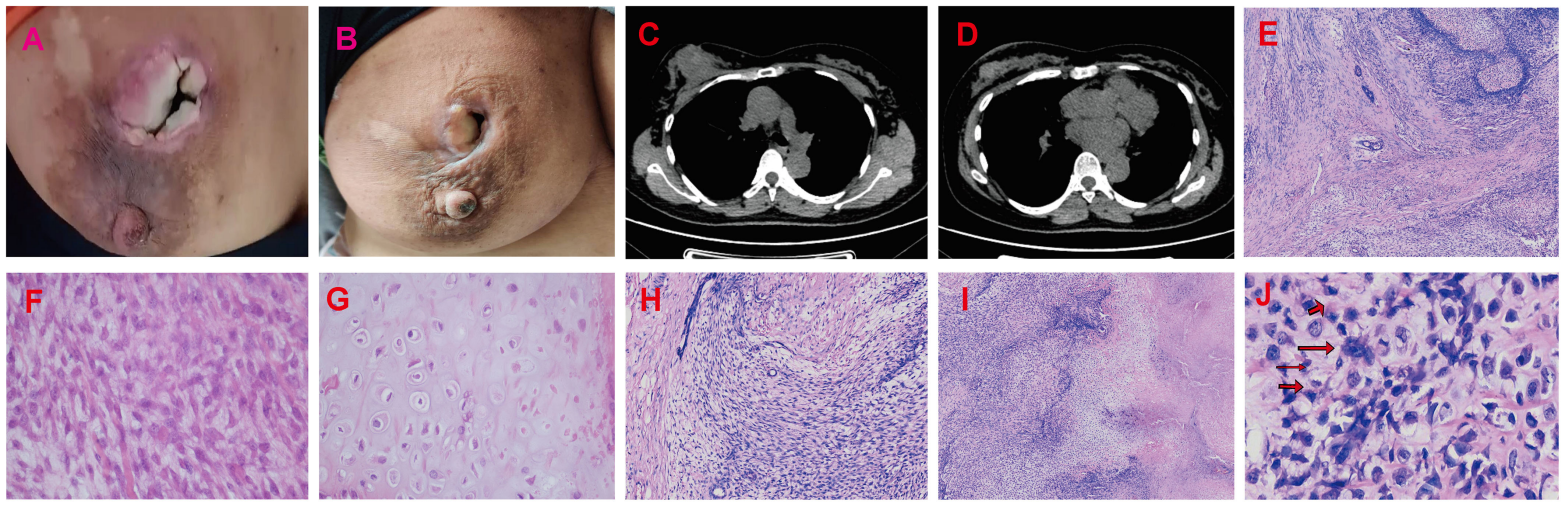
Radiotherapy and chemotherapy significantly reduced the size of the breast mass. **(A, B)** Graphic representation of breast lesion after radiotherapy and chemotherapy. **(C, D)** The CT images of the patient’s right breast after radiotherapy and chemotherapy. The H&E staining of Borderline Phyllodes Tumor with malignant features. **(E)** The benign epithelial components H&E×4. **(F)** The staining depicting of the spindle cell region H&E×400. **(G)** The staining illustrating the cartilage region H&E×400. **(H)** The permeative border of the borderline PT with malignant features H&E×100. **(I)** The interstitial overgrowth of borderline PT with malignant features H&E×40. **(J)** The nuclear division and pathological nuclear division H&E×400. H&E = hematoxylin and eosin.

**Figure 4:**
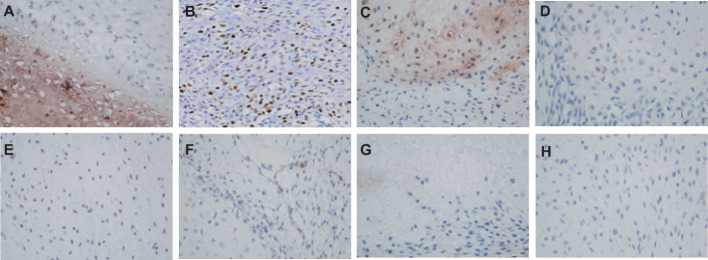
The IHC staining of Borderline Phyllodes Tumor with malignant features. **(A)** Strong positivity of ER×400. **(B)** A moderate level of Ki67 expression×200. **(C)** Weakly positivity of CD117 ×400. **(D)** Weakly positivity of P63 ×400. **(E)** Weakly positivity of Bcl2×400. **(F)** Negative staining of CD34×400. **(G)** Negative staining of CD10×400. **(H)** Negative staining of CK5/6 ×400. IHC, Immunohistochemistry.

The patient underwent neoadjuvant radiotherapy and chemotherapy, which included cytarabine and daunorubicin. After undergoing two cycles of chemotherapy, which included cytarabine at a dose of 100 mg/m2 administered over days 1-7, and daunorubicin at a dose of 60 mg/m2 over days 1-3, a follow-up CT scan revealed a significant reduction in the size of the breast mass (4cm×3cm) and a ground-glass appearance in the upper lobes of both lungs (**Figures 3A–D**). The patient presented with newly onset of dry cough and dyspnea. Empirical treatment with cefoperazone-tazobactam was initiated, but did not result in significant improvement of the patient’s condition. A fungal infection was suspected, and she was switched to voriconazole, which resolved her respiratory symptoms within 2 weeks. However, the patient declined subsequent cycles of chemotherapy due to adverse effects. Three months after completing chemotherapy, the patient underwent a right mastectomy. Histological analysis of the surgical specimen revealed the presence of both epithelial and mesenchymal components. Benign epithelial components are illustrated in **Figure 3E**. The spindle-shaped hyperproliferative fibroblasts, representative of the malignant components, are shown in **Figure 3F**. The cartilage region is considered to be heterogenous mesenchyme, which is associated with tumor invasion. (**Figure 3G**). Notably, these malignant interstitial cells are characterized by the permeative border (**Figure 3H**), heteromorphism and abundant mitosis, with a visible nuclear division rate of 11/10HPF (**Figures 3I, J**). In summary, the pathological analysis aligns with a diagnosis of borderline phyllodes tumor with malignant features. The IHC analysis demonstrated strong expression of ER (**Figure 4A**), PR, SMA and SATB2, with a moderate level of Ki67 expression (25-30%) (**Figure 4B**) and weak positivity for CD117 (**Figure 4C**) and Blc2 (**Figure 4D**). The tissues were negative for P63 (**Figure 4E**), CD34 (**Figure 4F**). CD10 (**Figure 4G**), desmin, cytokeratin-5/6 (**Figure 4H**).

The patient developed a local recurrence at the site of the surgical incision 4 months after the initial mastectomy and underwent another surgical excision to remove the new lesion. The subsequent pathological analysis confirmed the presence of borderline PT with malignant features. The patient has been closely monitored every 2 months since the second surgery and has remained disease-free for the past 8 months.

## 3 Discussion

MSs are rare tumors of immature myeloid cells that can occur outside the bone marrow, including the skin, lymph nodes, testes, intestines, bones, and central nervous system (3–7). Although MSs often develop concurrently with or after the diagnosis of myeloid malignancies (1, 8–10), cases of primary MS without any blood or bone marrow involvement (11–13), like our case, have also been reported. The rarity of MS can make diagnosing it challenging, leading to a higher incidence of misdiagnosis. Differential diagnoses of MS include various hematological malignancies such as anaplastic large-cell lymphoma and diffuse large B-cell lymphoma, as well as solid tumors such as breast carcinoma and melanoma (5, 13). Thus, a precise diagnosis of MS requires extensive immunophenotyping to confirm the myeloid origin of the malignancy. In our case, we observed the typical profile of myeloid malignant cells in the initial biopsy, which showed immunoreactivity to markers such as MPO (5) and CD33. Due to the rarity of myeloid sarcoma, there is currently no established consensus on the optimal treatment approach. Some previous studies have suggested that a combination of radiotherapy and chemotherapy may be beneficial for MS patients (14, 15), which is similar to our case. However, there is conflicting evidence, as another study reported no survival benefit in patients with isolated MS who were treated with radiation and chemotherapy (4).

PTs are considered an uncommon fibroepithelial neoplasms of the breast (16), which are classified into three subtypes: benign, borderline and malignant. The benign PTs are more common, An article analyzing 170 cases found that benign, borderline and malignant PTs incidence are 54.1%, 11.2% and 34.7% respectively (17). PTs pose diagnostic challenges, as they can be difficult to distinguish from fibroadenoma (18, 19). Histologically, PTs are characterized by a leaflike architecture resulting from an enhanced intracanalicular growth pattern, cleft-like spaces lined by epithelium, and hypercellular stroma. Malignant PT is characterized by marked stromal cellularity and nuclear pleomorphism, stromal overgrowth, and more than 10 mitoses per 10 HPF. The presence of heterologous sarcomatous elements (liposarcoma, chondrosarcoma, and osteosarcoma) alone qualifies a PT as malignant (20). In our case, the histological analysis revealed spindle cell and cartilage regions, with notable heteromorphism and mitosis in the spindle cell area. Additionally, there are reports indicating that the stromal cells of phyllodes tumors can express p63, cytokeratins and CD117 (21–23). Notably, CD117 expression in the stroma has been linked to predicting disease recurrence. In our patient, there were observed expressions of ER, PR, CD117, SMA, and SATB2, aligning with the previously documented trend as she experienced local recurrence.

To our knowledge, this is the first reported case of co-existing breast PT and MS. The co-occurrence posed further diagnostic challenges but also provides insights into the management of isolated MS. There is no definitive consensus regarding the optimal treatment and follow-up of primary extramedullary MS. Our patient benefited from neoadjuvant radiotherapy and chemotherapy, which led to a significant reduction in the breast mass and subsequent surgical excision with a clean margin. To our surprise, the repeated analysis of the surgical specimen revealed borderline PT with malignant features, without evidence of co-existing metastatic disease. We believe that the neoadjuvant therapies led to MS remission. This case highlights the importance of a comprehensive diagnostic approach and individualized treatment plan for patients with rare co-existing malignancies, such as PT and MS.

## 4 Conclusion

To our knowledge, this marks the inaugural report of a simultaneous occurrence of MS and PT in the breast. A prior study unveiled the coexistence of PT with anaplastic ependymoma (19), emphasizing the intricate nature of PT diagnosis when intertwined with other malignancies. Our case emphasizes the significance of adopting a comprehensive diagnostic approach to ensure precision, particularly in complex cases. The effective management of our patient’s condition with neoadjuvant radiotherapy and chemotherapy additionally underscores the potential advantages of this treatment strategy for individuals with myeloid sarcoma.

## Data availability statement

The original contributions presented in the study are included in the article/supplementary material. Further inquiries can be directed to the corresponding author.

## Ethics statement

Written informed consent was obtained from the individual(s) for the publication of any potentially identifiable images or data included in this article.

## Author contributions

LC: Resources, Writing – original draft. ZZ: Writing – review & editing, Methodology. QG: Writing – review & editing, Methodology. YH: Data curation, Writing – review & editing.
